# Supplementary material for: Experimental evidence that intruder and group member attributes affect outgroup defence and associated within-group interactions in a social fish
Source: Proc Biol Sci. 2019 Oct 9;286(1912):20191261. doi: 10.1098/rspb.2019.1261 (PMC6790772; doi:10.1098/rspb.2019.1261)
Supplement: Supplementary Methods [file rspb20191261supp1.docx]

**Experimental evidence that intruder and group member attributes affect outgroup defence and associated within-group interactions in a social fish**

Ines Braga Goncalves and Andrew N. Radford

http://dx.doi.org/10.1098/rspb.2019.1261

**Supplementary Methods**

*Study species and husbandry*

To minimise baseline aggression in our captive groups of *Neolamprologus pulcher*, we ensured that each dominant individual was at least 5 mm larger than the same-sex subordinate. Each tank (30 x 61 x 38 cm) was placed on a polystyrene sheet (10 mm thickness) to reduce disturbance effects from vibrations and noise. We kept tanks visually isolated from neighbouring tanks using flexible white ViPrint sheets (0.35 mm thickness) between them. Each tank contained 2–3 cm of sand (Sansibar river sand), a 75-W heater (Eheim), a filter (Eheim Ecco pro 130), a thermometer (Eheim), one (Experiment I) or two (Experiment II) flowerpot halves (10 cm wide) that served as shelters at the centre of the territory, and an artificial plant between the two shelters (Experiment II). Water temperature was maintained at 26.3 ± 0.2°C (mean ± s.e.) and room lights were set on a 13L:11D hour cycle (daylight from 7 am to 8 pm). Fish were fed twice daily: a combination of frozen brine shrimp, water fleas, prawns, mosquito larvae, mysid shrimp, bloodworms, cichlid diet, spirulina, copepods, krill and sludge worms in the mornings on Monday to Friday; and dry fish flakes in the evenings and weekends.

*Experimental protocol*

Prior to an experimental intrusion into a resident tank, we slid down one transparent and one opaque flexible partition (0.75 mm white ViPrint) through single-channel PVC tracks glued to the long walls, 8 cm from the tank edge. This created a side compartment at the edge of the territory of the resident group that had space for an intruder to move. An intruder, which had previously been netted out of its tank and held in a 1-L container for 10 min, was netted into the side compartment in the resident group tank and left to settle for another 5 min. Short handling times do not seem to affect behaviour adversely in this species, as individuals have been shown to resume normal social behaviour within 1–2 min of being fin-clipped [1,2]. Furthermore, after the 5-min settling time, our intruders were active, and spent most of the time swimming while facing the resident group during the simulated intrusions (intruder responsiveness mean ± s.e. (range), Experiment I: 79.7 ± 3.2% (21.9–99.7%); Experiment II: 86.3 ± 1.8% (56.2–98.9%)). At the end of the 10-min intrusion period, we replaced the opaque partition, netted the intruder out of the side compartment (out of sight from the resident group) and removed both partitions for a 10-min post-intrusion period. All intrusions were conducted in the mornings to minimise natural daily variations in behaviour, hormone levels and hunger [3].

In Experiment I, we used unfamiliar females of different sizes in the three treatments (Large, Medium, Small) to vary the level of threat posed to different group members. For each group: the Large intruder was size-matched to the dominant female (intruders differed in size by 0.2 ± 0.9 mm (mean ± s.e.) from the resident dominant females); the Medium intruder was size-matched to the subordinate female (intruders differed in size by 0.5 ± 0.7 mm from the subordinate females); and the Small intruder was smaller than both females (4.8 ± 0.6 mm and 16.7 ± 1.3 mm smaller than the subordinate and dominant females, respectively). Intruders were selected from other experimental and non-experimental social groups; individuals were not used as intruders in the same week that their group was used as the resident group.

In Experiment II, we used unfamiliar dominant females, matched in size to the resident dominant female (intruders differed in size from dominant females by 1.5 ± 1.1 mm) as intruders. Prior to an intrusion, we used a net to steer the subordinate female into a compartment of similar size and on the opposite side of the tank to that where the intrusion would take place and held her behind a transparent partition. During this period, the subordinate female could see her groupmates but not physically join them. Simultaneously, we slid down the partitions to create the intrusion compartment (see above). The intruder was then introduced and left to settle for 5 min as in Experiment I. No female was used as an intruder in more than one tank or in the same week that it was used as the resident dominant female.

Experiment I (n = 12 groups) was conducted between July and September 2017, and Experiment II (n = 14 groups) between November and December 2017. We measured the standard body lengths (from tip of nose to tip of caudal peduncle, mm) of all fish at the start of each experiment, using a measuring board with a 1 mm grid. In Experiment I, dominant males (DM) were 47.2 ± 1.9 mm (mean ± s.e.) long, dominant females (DF) were 47.3 ± 1.6 mm, subordinate females (SF) were 35.5 ± 0.7 mm, and subordinate males (SM) were 35.3 ± 0.7 mm. In Experiment II, DMs were 55.5 ± 2.2 mm long, DFs were 54.2 ± 1.3 mm, SFs were 41.0 ± 1.5 mm, and SMs were 40.0 ± 1.0 mm. All groups had been formed at least two weeks prior to the start of the experiments to allow them to settle into their territories. We checked tanks daily for signs of aggression and removed subordinates if they showed sustained submissive and avoidance behaviour towards the dominants or incurred physical injuries. In Experiment II, three subordinate males were removed from groups prior to the start of the experiment, and one subordinate female was removed following day two of intrusions due to increased levels of aggression received. We included the former three groups in the analyses, but excluded the latter from the experiment, resulting in a final sample size of 13 (10 groups of four individuals and three groups of three individuals).

For each trial period, we recorded the frequencies of three categories of within-group behaviour—aggression, submission and affiliation—displayed and received by each group member. As per previously established behavioural protocols for this species [4-6], aggression included ramming, biting, frontal approaches, fin and opercula spreading, and head-down displays; submission included hook displays, quivering and head-up postures; and affiliation included bumps, parallel swimming and following. We included bumps, also referred to as soft touches, in our affiliation category because, although it has been speculated that they may have submissive, conciliatory or aggressive functions [7], we have found no support for this in the literature; both ethograms for this species [4-6] and more recent articles on this species [8,9] have categorised bumps as an affiliative behaviour. Intruder responsiveness towards the resident group was recorded as the proportion of time intruders spent actively swimming whilst facing the resident group. Statistical analyses are based on 30 min of observations on each group member and on 10 min of observations on the intruders per trial.

*Statistical analyses*

We used linear mixed effects models (LMMs) for our analyses. The significance of fixed factors in our models was assessed by comparing a model including the factor of interest with a model excluding it and testing the change in deviance between the models with *X*^2^ tests [10]. We sequentially removed non-significant interactions followed by main fixed factors from the models using deviance tests until only factors that significantly improved the fit of the model were left. When an interaction was significant, we subdivided the dataset and analysed the effects of treatment on each individual category separately. We ran post-hoc paired t-tests to identify significant pairwise differences between treatments in behaviours displayed and received by all group members, and in the behaviours exchanged between DFs and SFs. We also ran 1-sample t-tests (against *zero*) to assess whether treatment effects on changes in behaviours exchanged between DFs and SFs represented departures from ‘no change’ in behaviour. All analyses were carried out in R-Studio [11, version 1.0.143] and SPSS 24 (IBM Statistics). Prior to formal analyses, we used the package *effects* [12, version 4.0-2] to inspect the fixed effects visually and plotted the residuals to confirm that their distribution conformed to linearity expectations. We used the function ‘lmer’ in the package *lme4* [13, version 1.1-17] to run all LMMs. The significance level (α) was set at 0.05 for all tests.

To assess how variation in intruder size (Experiment I) and how subordinate female presence or absence from the group and ability to assist during intrusions (Experiment II) affected defence behaviour of resident group members, we ran LMMs with a Gaussian error structure on square root-transformed frequencies of defensive acts against the intruder. We controlled for individual category, intruder responsiveness (covariate) and its possible interaction with treatment, and for trial order (fixed factor, Day 1–3). Group identity and subject identity (nested within group: Experiment I had 48 subjects, Experiment II had 49 subjects) were included as random factors to control for non-independence of subjects and for repeated observations on each subject. We did not control for intruder identity because only a small proportion of female intruders in Experiment I were used more than once, and no female in Experiment II was used as an intruder for more than one focal group. In Experiment II, we further controlled for group size and its potential interaction with treatment, because 10 groups had four members and three groups had only three members. Analyses considering within-group interactions during the intrusions in Experiment I included the same factors as the models on territorial defence. We did not assess within-group interactions during the intrusions in Experiment II because the Uncooperative treatment precluded the females from interacting to the same extent as in the Cooperative treatment, and the Unaware treatment prevented them from interacting entirely with the other group members. To investigate post-intrusion within-group interactions in both experiments, we calculated changes in frequencies of aggression, submission and affiliation displayed and received from the pre-intrusion to the post-intrusion period (no intruder was present in both periods). We controlled for individual category, trial order, group and subject identity. To study how DF–SF social interactions were impacted by the intrusions, we analysed the changes in frequencies of DF aggressive and affiliative behaviours directed at the SF, and on the changes in frequencies of SF affiliative and submissive behaviours directed at the DF, with treatment and trial order as fixed factors and subject identity as a random factor.

**References:**

[1] Stiver, K.A., Dierkes, P., Taborsky, M. & Balshine, S. 2004 Dispersal patterns and status change in a co-operatively breeding cichlid *Neolamprologus pulcher:* evidence from microsatellite analyses and behavioural observations. *J. Fish Biol.* **65**, 91–105.

[2] Mileva, V.R., Fitzpatrick, J.L., Marsh-Rollo, S., Gilmour, K.M., Wood, C.M. & Balshine, S. 2009 The stress response of the highly social African cichlid *Neolamprologus pulcher*. *Physiol. Biochem. Zool.* **82**, 720–729.

[3] Desjardins, J.K., Fitzpatrick, J.L., Stiver, K.A., Van Der Kraak, G.J. & Balshine, S. 2011 Lunar and diurnal cycles in reproductive physiology and behavior in a natural population of cooperatively breeding fish. *J. Zool.* **285**, 66–73.

[4] Taborsky, M. 1984 Broodcare helpers in the cichlid fish *Lamprologus brichardi* - their costs and benefits. *Anim. Behav.* **32**, 1236-1252.

[5] Sopinka, N.M., Fitzpatrick, J.L., Desjardins, J.K., Stiver, K.A., Marsh-Rollo, S.E. & Balshine, S. 2009 Liver size reveals social status in the African cichlid *Neolamprologus pulcher*. *J. Fish Biol.* **75**, 1-16.

[6] Reddon, A.R., O'Connor, C.M., Marsh-Rollo, S.E., Balshine, S., Gozdowska, M. & Kulczykowska, E. 2015 Brain nonapeptide levels are related to social status and affiliative behaviour in a cooperatively breeding cichlid fish. *R. Soc. Open Sci.* **2**, 140072.

[7] Hamilton, I.M., Heg, D. & Bender, N. 2005 Size differences within a dominance hierarchy influence conflict and help in a cooperatively breeding cichlid. *Behaviour* **142**, 1591-1613.

[8] Hellmann, J.K. & Hamilton, I.M. 2019 Intragroup social dynamics vary with the presence of neighbors in a cooperatively breeding fish. *Curr. Zool.* **65**, 21-31.

[9] Reyes-Contreras, M., Glauser, G., Rennison, D.J. & Taborsky, B. 2019 Early-life manipulation of cortisol and its receptor alters stress axis programming and social competence. *Philos. Trans. R. Soc. B-Biol. Sci.* **374**, 13.

[10] Bolker, B.M., Brooks, M.E., Clark, C.J., Geange, S.W., Poulsen, J.R., Stevens, M.H.H. & White, J.S.S. 2009 Generalized linear mixed models: a practical guide for ecology and evolution. *Trends Ecol. Evol.* **24**, 127–135.

[11] RStudio. 2016 Integrated Development Environment for R version 1.0.143. RStudio, Inc.

[12] Fox, J. & Weisberg, S. 2018 Visualizing fit and lack of fit in complex regression models with predictor effect plots and partial residuals. *J Stat Softw* **87**, 1–27.

[13] Bates, D., Maechler, M., B., B. & Walker, S. 2015 Fitting Linear Mixed-Effects Models Using lme4. *J Stat Softw* **67**, 1-48.
