# Supplementary material for: Experimental evidence that intruder and group member attributes affect outgroup defence and associated within-group interactions in a social fish
Source: Proc Biol Sci. 2019 Oct 9;286(1912):20191261. doi: 10.1098/rspb.2019.1261 (PMC6790772; doi:10.1098/rspb.2019.1261)
Supplement: Supplementary Results [file rspb20191261supp2.docx]

**Experimental evidence that intruder and group member attributes affect outgroup defence and associated within-group interactions in a social fish**

Ines Braga Goncalves and Andrew N. Radford

http://dx.doi.org/10.1098/rspb.2019.1261

**Supplementary Results**

Results of linear mixed models investigating behaviour in response to experimental intrusions. In both experiments, Individual category is a composite measure of individual sex and social rank, with four possible levels: dominant female (DF), dominant male (DM), subordinate female (SF) and subordinate male (SM). In Experiment I, Treatment relates to intruder size (Small, Medium and Large). In Experiment II, Treatment relates to whether the SF in the group was able to observe the intruder and participate in territorial defence (Cooperative), could observe the intruder but not participate in territorial defence (Uncooperative), or could neither observe nor participate in territorial defence (Unaware). In each experiment, because trials were run on three consecutive days in a counterbalanced order, Order (Day 1, Day 2 or Day 3) was controlled for in the statistical models. In Experiment II, Group size (3 or 4 group members) was also included. Intruder responsiveness (the proportion of time the intruder was active and facing the resident group during the intrusion period) was included in all models assessing resident group behaviour during intrusions. The interaction between Individual category and Treatment was assessed in all models of within-group behaviour and changes in behaviour; the interaction between treatment and Intruder responsiveness was assessed in all models of within-group behaviour displayed during intrusions; the interaction between Treatment and Group size was assessed in all models run on Experiment II datasets. All models contained either Subject and Group as nested random terms or just Subject as a random term (with variance ± s.d. shown). For fixed effects included in significant interactions, only parameter estimates are shown. Non-significant fixed terms are shown by order of removal. The reference level for Treatment was either Large intruder (Experiment I) or Cooperative (Experiment II), for Individual category was DF, for Order was Day 1, and for Group size was three. Significant terms are shown in bold. tables S1 and S2 relate to territorial defence behaviour (Experiments I and II); tables S3 to S6 relate to within-group interactions during intrusions (Experiment I); and tables S7 to S14 relate to post-intrusion within-group changes in behaviour (Experiments I and II).

**table S1** – Effect of female intruder size (Experiment I) on defence behaviour of resident group members (square-root transformed number of aggressive acts directed at the intruder).

| Subject: Group: 4.08 ± 2.02; Group: 0.00 ± 0.00; Residual: 2.65 ± 1.63 | | | | | | |
| --- | --- | --- | --- | --- | --- | --- |
| FINAL MODEL | *Χ*^2^ | t-value | d.f. | p | estimate | s.e. |
| **Intercept** |  | **8.19** | **75.67** | **<0.001** | **6.14** | **0.75** |
| Treatment |  |  |  |  |  |  |
| *Medium intruder* |  |  |  |  | -1.52 | 0.66 |
| *Small intruder* |  |  |  |  | -2.17 | 0.66 |
| Individual category |  |  |  |  |  |  |
| *DM* |  |  |  |  | -1.79 | 1.06 |
| *SF* |  |  |  |  | -2.92 | 1.06 |
| *SM* |  |  |  |  | -2.65 | 1.06 |
| **Treatment x Individual category** | **37.6** |  | **6** | **<0.001** |  |  |
| Medium intruder x Individual category |  |  |  |  |  |  |
| *DM* |  | 1.88 | 88 | 0.064 | 1.76 | 0.94 |
| ***SF*** |  | **5.71** | **88** | **<0.001** | **5.35** | **0.94** |
| ***SM*** |  | **2.69** | **88** | **0.009** | **2.52** | **0.94** |
| Small intruder x Individual category |  |  |  |  |  |  |
| *DM* |  | 1.92 | 88 | 0.058 | 1.80 | 0.94 |
| ***SF*** |  | **4.95** | **88** | **<0.001** | **4.64** | **0.94** |
| ***SM*** |  | **2.75** | **88** | **0.007** | **2.57** | **0.94** |
| REMOVED TERMS | *Χ*^2^ |  | d.f. | p |  |  |
| Intruder responsiveness x Treatment | 2.12 |  | 2 | 0.347 |  |  |
| Intruder responsiveness | 1.05 |  | 1 | 0.305 |  |  |
| Order | 3.76 |  | 2 | 0.152 |  |  |

**table S2** – Effect of subordinate female ability to observe the intrusion and/or participate in territorial defence (Experiment II) on defence behaviour of resident group members (square-root transformed number of aggressive acts directed at the intruder).

| Subject: Group: 0.84 ± 0.91; Group: 0.05 ± 0.22; Residual: 1.01 ± 1.00 | | | | | | |
| --- | --- | --- | --- | --- | --- | --- |
| FINAL MODEL | *Χ*^2^ | t-value | d.f. | p | estimate | s.e. |
| **Intercept** |  | **12.62** | **28.88** | **<0.001** | **5.74** | **0.46** |
| **Individual category** | **68.9** |  | **3** | **<0.001** |  |  |
| ***DM*** |  | **-2.49** | **33.58** | **0.018** | **-1.06** | **0.43** |
| ***SF*** |  | **-10.35** | **33.58** | **<0.001** | **-4.40** | **0.43** |
| ***SM*** |  | **-8.07** | **33.58** | **<0.001** | **-3.76** | **0.47** |
| **Group size** | **9.57** |  | **1** | **0.002** |  |  |
| ***Four individuals*** |  | **-3.31** | **17.19** | **0.004** | **-1.44** | **0.44** |
| REMOVED TERMS | *Χ*^2^ |  | d.f. | p |  |  |
| Intruder responsiveness x Treatment | 0.68 |  | 2 | 0.714 |  |  |
| Treatment x Group Size | 3.30 |  | 2 | 0.192 |  |  |
| Treatment x Individual category | 7.36 |  | 6 | 0.289 |  |  |
| Intruder responsiveness | 0.02 |  | 1 | 0.900 |  |  |
| Order | 1.02 |  | 2 | 0.601 |  |  |
| Treatment | 0.41 |  | 2 | 0.816 |  |  |

**table S3** – Effect of female intruder size (Experiment I) on number of aggressive behaviours (a) displayed at group members and (b) received from group members, during intrusions.

| **a. Within-group aggression displayed** | | | | | | |
| --- | --- | --- | --- | --- | --- | --- |
| Subject: Group: 13.94 ± 3.73; Group: 0.00 ± 0.00; Residual: 35.96 ± 6.00 | | | | | | |
| FINAL MODEL | *Χ*^2^ | t-value | d.f. | p | estimate | s.e. |
| **Intercept** |  | **6.62** | **65.07** | **<0.001** | **10.80** | **1.63** |
| **Treatment** | **7.94** |  | **2** | **0.019** |  |  |
| ***Medium intruder*** |  | **-2.83** | **94.00** | **0.006** | **-3.46** | **1.22** |
| *Small intruder* |  | -1.11 | 94.00 | 0.271 | -1.35 | 1.22 |
| **Individual category** | **17.70** |  | **3** | **<0.001** |  |  |
| *DM* |  | -1.36 | 44.00 | 0.180 | -2.83 | 2.08 |
| ***SF*** |  | **-4.21** | **44.00** | **<0.001** | **-8.75** | **2.08** |
| ***SM*** |  | **-2.74** | **44.00** | **0.009** | **-5.69** | **2.08** |
| REMOVED TERMS | *Χ*^2^ |  | d.f. | p |  |  |
| Intruder responsiveness x Treatment | 0.63 |  | 2 | 0.730 |  |  |
| Treatment x Individual category | 11.99 |  | 6 | 0.062 |  |  |
| Intruder responsiveness | 0.05 |  | 1 | 0.822 |  |  |
| Order | 5.32 |  | 2 | 0.070 |  |  |
| **b. Within-group aggression received** | | | | | | |
| Subject: Group: 16.09 ±4.01; Group: 0.00 ± 0.00; Residual: 41.27 ± 6.42 | | | | | | |
| FINAL MODEL | *Χ*^2^ | t-value | d.f. | p | estimate | s.e. |
| Intercept |  | 0.65 | 114.05 | 0.518 | 1.42 | 2.19 |
| Treatment |  |  |  |  |  |  |
| *Medium intruder* |  |  |  |  | -0.25 | 2.62 |
| *Small intruder* |  |  |  |  | 0.58 | 2.62 |
| Individual category |  |  |  |  |  |  |
| *DM* |  |  |  |  | 0.42 | 3.09 |
| *SF* |  |  |  |  | 16.75 | 3.09 |
| *SM* |  |  |  |  | 2.33 | 3.09 |
| **Treatment x Individual category** | **18.27** |  | **6** | **0.006** |  |  |
| Medium intruder x Individual category |  |  |  |  |  |  |
| *DM* |  | 0.29 | 88.00 | 0.771 | 1.08 | 3.71 |
| ***SF*** |  | **-3.26** | **88.00** | **0.002** | **-12.08** | **3.71** |
| *SM* |  | -0.29 | 88.00 | 0.771 | 1.08 | 3.71 |
| Small intruder x Individual category |  |  |  |  |  |  |
| *DM* |  | -0.16 | 88.00 | 0.875 | -0.58 | 3.71 |
| ***SF*** |  | **-2.31** | **88.00** | **0.023** | **-8.58** | **3.71** |
| *SM* |  | 0.40 | 88.00 | 0.687 | 1.50 | 3.71 |
| REMOVED TERMS | *Χ*^2^ |  | d.f. | p |  |  |
| Intruder responsiveness x Treatment | 0.61 |  | 2 | 0.736 |  |  |
| Intruder responsiveness | 0.06 |  | 1 | 0.802 |  |  |
| Order | 4.8 |  | 2 | 0.091 |  |  |

**table S4** – Effect of female intruder size (Experiment I) on number of submissive behaviours (a) displayed at group members and (b) received from group members, during intrusions.

| **a. Within-group submission displayed** | | | | | | |
| --- | --- | --- | --- | --- | --- | --- |
| Subject: Group: 2.07 ± 1.44; Group: 0.00 ± 0.00; Residual: 6.67 ± 2.58 | | | | | | |
| FINAL MODEL | *Χ*^2^ | t-value | d.f. | p | estimate | s.e. |
| **Intercept** |  | **3.02** | **67.64** | **0.004** | **2.03** | **0.67** |
| **Individual category** | **20.17** |  | **3** | **<0.001** |  |  |
| ***DM*** |  | **2.76** | **44.00** | **0.008** | **2.33** | **0.85** |
| ***SF*** |  | **3.61** | **44.00** | **<0.001** | **3.06** | **0.85** |
| ***SM*** |  | **4.53** | **44.00** | **<0.001** | **3.83** | **0.85** |
| **Order** | **11.8** |  | **2** | **0.003** |  |  |
| ***Day 2*** |  | **-2.09** | **94.00** | **0.039** | **-1.10** | **0.53** |
| ***Day 3*** |  | **-3.44** | **94.00** | **<0.001** | **-1.81** | **0.53** |
| REMOVED TERMS | *Χ*^2^ |  | d.f. | p |  |  |
| Intruder responsiveness x Treatment | 3.86 |  | 2 | 0.145 |  |  |
| Treatment x Individual category | 8.93 |  | 6 | 0.178 |  |  |
| Intruder responsiveness | 0.04 |  | 1 | 0.843 |  |  |
| Treatment | 2.26 |  | 2 | 0.323 |  |  |
| **b. Within-group submission received** | | | | | | |
| Subject: Group: 3.59 ± 1.90; Group: 0.00 ± 0.00; Residual: 7.09 ± 2.66 | | | | | | |
| FINAL MODEL |  | t-value | d.f. | p | estimate | s.e. |
| **Intercept** |  | **11.30** | **62.05** | **<0.001** | **8.71** | **0.77** |
| **Individual category** | **45.84** |  | **3** | **<0.001** |  |  |
| ***DM*** |  | **-3.35** | **44.00** | **0.002** | **-3.33** | **1.00** |
| ***SF*** |  | **-7.11** | **44.00** | **<0.001** | **-7.08** | **1.00** |
| ***SM*** |  | **-7.11** | **44.00** | **<0.001** | **-7.08** | **1.00** |
| **Order** | **11.22** |  | **2** | **0.004** |  |  |
| ***Day 2*** |  | **-2.11** | **94.00** | **0.038** | **-1.15** | **0.54** |
| ***Day 3*** |  | **-3.34** | **94.00** | **0.001** | **-1.81** | **0.54** |
| REMOVED TERMS | *Χ*^2^ |  | d.f. | p |  |  |
| Intruder responsiveness x Treatment | 3.6 |  | 2 | 0.17 |  |  |
| Treatment x Individual category | 1.67 |  | 6 | 0.95 |  |  |
| Intruder responsiveness | 0.19 |  | 1 | 0.67 |  |  |
| Treatment | 2.18 |  | 2 | 0.34 |  |  |

**table S5** – Effect of female intruder size (Experiment I) on number of affiliative behaviours (a) displayed at group members and (b) received from group members, during intrusions.

| **a. Within-group affiliation displayed** | | | | | | |
| --- | --- | --- | --- | --- | --- | --- |
| Subject: Group: 11.71 ± 3.42; Group: 7.69 ± 2.77; Residual: 10.92 ± 3.31 | | | | | | |
| FINAL MODEL | *Χ*^2^ | t-value | d.f. | p | estimate | s.e. |
| **Intercept** |  | **4.65** | **32.98** | **<0.001** | **6.44** | **1.39** |
| **Individual category** | **10.18** |  | **3** | **0.017** |  |  |
| *DM* |  | 1.44 | 33.00 | 0.159 | 2.31 | 1.60 |
| *SF* |  | -1.81 | 33.00 | 0.080 | -2.89 | 1.60 |
| *SM* |  | -0.49 | 33.00 | 0.630 | -0.78 | 1.60 |
| REMOVED TERMS | *Χ*^2^ |  | d.f. | p |  |  |
| Intruder responsiveness x Treatment | 5.57 |  | 2 | 0.083 |  |  |
| Treatment x Individual category | 7.42 |  | 6 | 0.284 |  |  |
| Intruder responsiveness | 0.52 |  | 1 | 0.472 |  |  |
| Order | 3.33 |  | 2 | 0.190 |  |  |
| Treatment | 2.87 |  | 2 | 0.238 |  |  |
| **b. Within-group affiliation received** | | | | | | |
| Subject: Group: 5.46 ± 2.34; Group: 9.48 ± 3.08; Residual: 8.63 ± 2.94 | | | | | | |
| FINAL MODEL | *Χ*^2^ | t-value | d.f. | p | estimate | s.e. |
| **Intercept** |  | **7.20** | **23.78** | **<0.001** | **8.78** | **1.22** |
| **Individual category** | **20.14** |  | **3** | **<0.001** |  |  |
| *DM* |  | -1.18 | 33.00 | 0.247 | -1.39 | 1.18 |
| ***SF*** |  | **-4.31** | **33.00** | **<0.001** | **-5.08** | **1.18** |
| ***SM*** |  | **-3.63** | **33.00** | **<0.001** | **-4.28** | **1.18** |
| REMOVED TERMS | *Χ*^2^ |  | d.f. | p |  |  |
| Intruder responsiveness x Treatment | 4.08 |  | 2 | 0.211 |  |  |
| Treatment x Individual category | 6.1 |  | 6 | 0.412 |  |  |
| Intruder responsiveness | 0.84 |  | 1 | 0.359 |  |  |
| Order | 4.52 |  | 2 | 0.104 |  |  |
| Treatment | 3.49 |  | 2 | 0.174 |  |  |

**table S6 -** Effect of female intruder size (Experiment I) on number of (a) DF aggressive behaviours directed at the SF, (b) SF submissive behaviours directed at the DF, (c) DF affiliative behaviours directed at the SF, and (d) SF affiliative behaviours directed at the DF, during intrusions.

| **a. DF aggression directed at SF** |  |  |  |  |  |  |
| --- | --- | --- | --- | --- | --- | --- |
| Subject: 17.35 ± 4.17; Residual: 19.58 ± 4.43 | | | | | | |
| FINAL MODEL | *Χ*^2^ | t-value | d.f. | p | estimate | s.e. |
| **Intercept** |  | **3.58** | **11.00** | **0.004** | **5.06** | **1.41** |
| REMOVED TERMS | *Χ*^2^ |  | d.f. | p |  |  |
| Intruder responsiveness x Treatment | 0.62 |  | 2 | 0.734 |  |  |
| Intruder responsiveness | 0.64 |  | 1 | 0.424 |  |  |
| Order | 1.29 |  | 2 | 0.524 |  |  |
| Treatment | 4.75 |  | 2 | 0.093 |  |  |
| **b. SF submission directed at DF** |  |  |  |  |  |  |
| Subject: 0.12 ± 0.34; Residual: 4.14 ± 2.03 | | | | | | |
| FINAL MODEL | *Χ*^2^ | t-value | d.f. | p | estimate | s.e. |
| **Intercept** |  | **11.30** | **62.05** | **<0.001** | **8.71** | **0.77** |
| REMOVED TERMS | *Χ*^2^ |  | d.f. | p |  |  |
| Intruder responsiveness x Treatment | 5.73 |  | 2 | 0.057 |  |  |
| Intruder responsiveness | 0.25 |  | 1 | 0.618 |  |  |
| Order | 2.88 |  | 2 | 0.237 |  |  |
| Treatment | 2.41 |  | 2 | 0.299 |  |  |
| **c. DF affiliation directed at SF** |  |  |  |  |  |  |
| Subject: 2.89 ± 1.70; Residual: 0.40 ± 0.62 | | | | | | |
| FINAL MODEL | *Χ*^2^ | t-value | d.f. | p | estimate | s.e. |
| **Intercept** |  | **2.67** | **29.99** | **0.012** | **2.32** | **0.87** |
| Treatment |  |  |  |  |  |  |
| *Medium Intruder* |  |  |  |  | -1.48 | 2.21 |
| *Small Intruder* |  |  |  |  | -3.27 | 1.14 |
| Intruder responsiveness |  |  |  |  | -1.73 | 0.91 |
| **Intruder responsiveness x Intruder** | **10.33** |  | **2** | **0.006** |  |  |
| *Intruder responsiveness x Medium Intruder* |  | 0.80 | 19.93 | 0.435 | 1.20 | 2.51 |
| ***Intruder responsiveness x Small Intruder*** |  | **3.07** | **19.62** | **0.006** | **4.60** | **1.50** |
| REMOVED TERMS | *Χ*^2^ |  | d.f. | p |  |  |
| Order | 0.92 |  | 2 | 0.631 |  |  |
| **d. SF affiliation directed at DF** |  |  |  |  |  |  |
| Subject: 1.42 ± 1.19; Residual: 3.12 ± 1.77 | | | | | | |
| FINAL MODEL | *Χ*^2^ | t-value | d.f. | p | estimate | s.e. |
| **Intercept** |  | **5.78** | **33.90** | **<0.001** | **8.74** | **1.51** |
| **Intruder responsiveness** | **16.36** |  | **1** | **<0.001** |  |  |
| ***Intruder responsiveness*** |  | **-4.90** | **33.73** | **<0.001** | **-8.88** | **1.81** |
| REMOVED TERMS | *Χ*^2^ |  | d.f. | p |  |  |
| Intruder responsiveness x Intruder | 3.80 |  | 2 | 0.150 |  |  |
| Order | 0.35 |  | 2 | 0.840 |  |  |
| Treatment | 2.00 |  | 2 | 0.370 |  |  |

**table S7** – Effect of female intruder size (Experiment I) on post-intrusion changes in the number of aggressive behaviours (a) displayed at group members and (b) received from group members.

| **a. Change in within-group aggression displayed** | | |  |  |  |  |
| --- | --- | --- | --- | --- | --- | --- |
| Subject: Group: 67.16 ± 2.68; Group: 2.43 ± 1.56; Residual: 57.31 ± 7.57 | | | | | | |
| FINAL MODEL | *Χ*^2^ | t-value | d.f. | p | estimate | s.e. |
| Intercept |  | 2.02 | 11.00 | 0.068 | 1.75 | 0.87 |
| REMOVED TERMS | *Χ*^2^ |  | d.f. | p |  |  |
| Treatment x Individual category | 3.77 |  | 6 | 0.708 |  |  |
| Order | 1.02 |  | 2 | 0.602 |  |  |
| Treatment | 1.14 |  | 2 | 0.565 |  |  |
| Individual category | 6.37 |  | 3 | 0.095 |  |  |
| **b. Change in within-group aggression received** | |  |  |  |  |  |
| Subject: Group: 23.51 ± 4.85; Group: 0.00 ± 0.00; Residual: 37.31 ± 6.11 | | | | | | |
| FINAL MODEL | *Χ*^2^ | t-value | d.f. | p | estimate | s.e. |
| **Intercept** |  | **2.10** | **47.00** | **0.040** | **1.82** | **0.87** |
| REMOVED TERMS | *Χ*^2^ |  | d.f. | p |  |  |
| Treatment x Individual category | 6.1 |  | 6 | 0.412 |  |  |
| Order | 2.03 |  | 2 | 0.362 |  |  |
| Treatment | 1.79 |  | 2 | 0.409 |  |  |
| Individual category | 5.54 |  | 3 | 0.137 |  |  |

**table S8** – Effect of female intruder size (Experiment I) on post-intrusion changes in the number of submissive behaviours (a) displayed at group members and (b) received from group members.

| **a. Change in within-group submission displayed** | | |  |  |  |  |
| --- | --- | --- | --- | --- | --- | --- |
| Subject: Group: 0.00 ± 0.00; Group: 0.00 ± 0.00; Residual: 16.39 ± 4.05 | | | | | | |
| FINAL MODEL | *Χ*^2^ | t-value | d.f. | p | estimate | s.e. |
| Intercept |  | 1.36 | 143.00 | 0.176 | 0.46 | 0.34 |
| REMOVED TERMS | *Χ*^2^ |  | d.f. | p |  |  |
| Treatment x Individual category | 6.24 |  | 6 | 0.397 |  |  |
| Order | 0.53 |  | 2 | 0.768 |  |  |
| Individual category | 3.91 |  | 3 | 0.271 |  |  |
| Treatment | 5.23 |  | 2 | 0.073 |  |  |
| **b. Change in within-group submission received** | |  |  |  |  |  |
| Subject: Group: 0.00 ± 0.00; Group: 0.00 ± 0.00; Residual: 17.20 ± 4.15 | | | | | | |
| FINAL MODEL | *Χ*^2^ | t-value | d.f. | p | estimate | s.e. |
| Intercept |  | 1.33 | 143.00 | 0.187 | 0.46 | 0.35 |
| REMOVED TERMS | *Χ*^2^ |  | d.f. | p |  |  |
| Treatment x Individual category | 4.16 |  | 6 | 0.655 |  |  |
| Order | 0.50 |  | 2 | 0.780 |  |  |
| Individual category | 2.78 |  | 3 | 0.428 |  |  |
| Treatment | 4.98 |  | 2 | 0.083 |  |  |

**table S9** – Effect of female intruder size (Experiment I) on post-intrusion changes in the number of affiliative behaviours (a) displayed at group members and (b) received from group members.

| **a. Change in within-group affiliation displayed** | |  |  |  |  |  |
| --- | --- | --- | --- | --- | --- | --- |
| Subject: Group: 0.00 ± 0.00; Group: 3.59 ± 1.89; Residual: 32.28 ± 5.68 | | | | | | |
| FINAL MODEL | *Χ*^2^ | t-value | d.f. | p | estimate | s.e. |
| Intercept |  | 0.17 | 11.00 | 0.866 | 0.13 | 0.72 |
| REMOVED TERMS | *Χ*^2^ |  | d.f. | p |  |  |
| Treatment x Individual category | 4.19 |  | 6 | 0.651 |  |  |
| Order | 3.11 |  | 2 | 0.211 |  |  |
| Individual category | 0.84 |  | 3 | 0.839 |  |  |
| Treatment | 0.97 |  | 2 | 0.615 |  |  |
| **b. Change in within-group affiliation received** | |  |  |  |  |  |
| Subject: Group: 0.00 ± 0.00; Group:3.14 ± 1.77; Residual: 37.67 ± 6.14 | | | | | | |
| FINAL MODEL | *Χ*^2^ | t-value | d.f. | p | estimate | s.e. |
| Intercept |  | 0.17 | 11.00 | 0.866 | 0.13 | 0.72 |
| REMOVED TERMS | *Χ*^2^ |  | d.f. | p |  |  |
| Treatment x Individual category | 8.61 |  | 6 | 0.197 |  |  |
| Order | 2.74 |  | 2 | 0.255 |  |  |
| Treatment | 0.86 |  | 2 | 0.650 |  |  |
| Individual category | 4.4 |  | 3 | 0.221 |  |  |

**table S10 -** Effect of female intruder size (Experiment I) on changes in post-intrusion (a) DF aggressive behaviours directed at the SF, (b) SF submissive behaviours directed at the DF, (c) DF affiliative behaviours directed at the SF, and (d) SF affiliative behaviours directed at the DF.

| **a. Change in DF aggression directed at SF** | | | | | | |
| --- | --- | --- | --- | --- | --- | --- |
| Subject: 10.08 ± 3.18; Residual: 100.08 ± 10.00 | | | | | | |
| FINAL MODEL | *Χ*^2^ | t-value | d.f. | p | estimate | s.e. |
| Intercept |  | 0.06 | 11.00 | 0.954 | 0.11 | 1.91 |
| REMOVED TERMS | *Χ*^2^ |  | d.f. | p |  |  |
| Treatment | 0.53 |  | 2 | 0.769 |  |  |
| Order | 1.05 |  | 2 | 0.591 |  |  |
| **b. Change in DF affiliation directed at SF** | | | | | | |
| Subject: 0.00 ± 0.00; Residual: 7.03 ± 2.65 | | | | | | |
| FINAL MODEL | *Χ*^2^ | t-value | d.f. | p | estimate | s.e. |
| Intercept |  | -0.13 | 35.00 | 0.901 | -0.06 | 0.44 |
| REMOVED TERMS | *Χ*^2^ |  | d.f. | p |  |  |
| Treatment | 0.11 |  | 2 | 0.948 |  |  |
| Order | 1.66 |  | 2 | 0.436 |  |  |
| **c. Change in SF submission directed at DF** | | | | | | |
| Subject: 0.00 ± 0.00; Residual:13.82 ± 3.72 | | | | | | |
| FINAL MODEL | *Χ*^2^ | t-value | d.f. | p | estimate | s.e. |
| Intercept |  | 1.30 | 35.00 | 0.202 | 0.81 | 0.62 |
| REMOVED TERMS | *Χ*^2^ |  | d.f. | p |  |  |
| Order | 1.96 |  | 2 | 0.375 |  |  |
| Treatment | 2.02 |  | 2 | 0.364 |  |  |
| **d. Change in SF affiliation directed at DF** | | | | | | |
| Subject: 0.00 ± 0.00; Residual: 11.19 ± 3.35 | | | | | | |
| FINAL MODEL | *Χ*^2^ | t-value | d.f. | p | estimate | s.e. |
| Intercept |  | -0.19 | 33.00 | 0.852 | -0.17 | 0.89 |
| **Treatment** | **8.35** |  | **2** | **0.015** |  |  |
| *Medium intruder* |  | -0.87 | 33.00 | 0.393 | -1.08 | 1.25 |
| *Small intruder* |  | 2.00 | 33.00 | 0.054 | 2.50 | 1.25 |
| REMOVED TERMS | *Χ*^2^ |  | d.f. | p |  |  |
| Order | 4.18 |  | 2 | 0.124 |  |  |

**table S11** – – Effect of subordinate female ability to observe the intrusion and/or participate in territorial defence (Experiment II) on post-intrusion changes in number of aggressive behaviours (a) displayed at group members and (b) received from group members.

| **a. Change in within-group aggression displayed** | |  |  |  |  |  |
| --- | --- | --- | --- | --- | --- | --- |
| Subject: Group: 0.00 ± 0.00; Group: 2.28 ± 1.51; Residual: 21.52 ± 4.64 | | | | | | |
| FINAL MODEL | *Χ*^2^ | t-value | d.f. | p | estimate | s.e. |
| Intercept |  | 1.54 | 12.27 | 0.150 | 0.87 | 0.57 |
| REMOVED TERMS | *Χ*^2^ |  | d.f. | p |  |  |
| Group size x Treatment | 2.66 |  | 2 | 0.264 |  |  |
| Treatment x Individual category | 7.88 |  | 6 | 0.247 |  |  |
| Group size | 2.59 |  | 1 | 0.107 |  |  |
| Order | 3.21 |  | 2 | 0.201 |  |  |
| Individual category | 5.93 |  | 3 | 0.115 |  |  |
| Treatment | 0.66 |  | 2 | 0.719 |  |  |
| **b. Change in within-group aggression received** | | | | | | |
| Subject: Group: 0.16 ±0.40; Group: 2.62 ± 1.62; Residual: 13.64 ± 3.69 | | | | | | |
| FINAL MODEL | *Χ*^2^ | t-value | d.f. | p | estimate | s.e. |
| Intercept |  | -1.30 | 33.56 | 0.203 | -0.97 | 0.75 |
| **Individual category** | **27.13** |  | **3** | **<0.001** |  |  |
| *DM* |  | 0.24 | 33.37 | 0.811 | 0.21 | 0.85 |
| ***SF*** |  | **4.64** | **33.37** | **<0.001** | **3.95** | **0.85** |
| ***SM*** |  | **4.04** | **34.32** | **<0.001** | **3.74** | **0.93** |
| REMOVED TERMS | *Χ*^2^ |  | d.f. | p |  |  |
| Group size x Treatment | 2.49 |  | 2 | 0.287 |  |  |
| Treatment x Individual category | 6.23 |  | 6 | 0.398 |  |  |
| Group size | 0.57 |  | 1 | 0.451 |  |  |
| Order | 5.04 |  | 2 | 0.080 |  |  |
| Treatment | 1.16 |  | 2 | 0.560 |  |  |

**table S12** – – Effect of subordinate female ability to observe the intrusion and/or participate in territorial defence (Experiment II) on post-intrusion changes in number of submissive behaviours (a) displayed at group members and (b) received from group members.

| **a. Change in within-group submission displayed** | | | | | | |
| --- | --- | --- | --- | --- | --- | --- |
| Subject: Group: 3.55 ± 1.88; Group: 6.19 ± 2.49; Residual: 41.18 ± 6.42 | | | | | | |
| FINAL MODEL | *Χ*^2^ | t-value | d.f. | p | estimate | s.e. |
| Intercept |  | 0.33 | 62.64 | 0.744 | 0.51 | 1.54 |
| **Treatment** | **7.29** |  | **2** | **0.026** |  |  |
| ***Uncooperative*** |  | **-2.71** | **96.00** | **0.008** | **-3.51** | **1.30** |
| *Unaware* |  | -1.61 | 96.00 | 0.112 | -2.08 | 1.30 |
| **Individual category** | **14.07** |  | **3** | **0.003** |  |  |
| *DM* |  | -0.87 | 33.27 | 0.393 | -1.41 | 1.63 |
| ***SF*** |  | **2.25** | **33.27** | **0.031** | **3.67** | **1.63** |
| ***SM*** |  | **2.40** | **34.48** | **0.022** | **4.24** | **1.77** |
| REMOVED TERMS | *Χ*^2^ |  | d.f. | p |  |  |
| Group size x Treatment | 4.19 |  | 2 | 0.123 |  |  |
| Treatment x Individual category | 9.41 |  | 6 | 0.152 |  |  |
| Group size | 0.03 |  | 1 | 0.871 |  |  |
| Order | 5.57 |  | 2 | 0.062 |  |  |
| **b. Change in within-group submission received** | | | | | | |
| Subject: Group: 26.87 ± 5.18; Group: 0.00 ± 0.00; Residual: 48.10 ± 6.94 | | | | | | |
| FINAL MODEL | *Χ*^2^ | t-value | d.f. | p | estimate | s.e. |
| **Intercept** |  | **4.04** | **110.01** | **<0.001** | **7.92** | **1.96** |
| Treatment |  |  |  |  |  |  |
| *Uncooperative* |  |  |  |  | -9.85 | 2.42 |
| *Unaware* |  |  |  |  | -7.69 | 2.42 |
| Individual category |  |  |  |  |  |  |
| *DM* |  |  |  |  | -8.85 | 2.61 |
| *SF* |  |  |  |  | -7.77 | 2.61 |
| *SM* |  |  |  |  | -7.70 | 2.81 |
| **Treatment x Individual category** | **13.41** |  | **6** | **0.037** |  |  |
| Uncooperative treatment x Individual category | | |  |  |  |  |
| ***DM*** |  | **2.27** | **90.00** | **0.026** | **7.77** | **3.43** |
| ***SF*** |  | **2.61** | **90.00** | **0.011** | **8.92** | **3.43** |
| ***SM*** |  | **2.55** | **90.00** | **0.013** | **9.35** | **3.67** |
| Unaware treatment x Individual category | |  |  |  |  |  |
| ***DM*** |  | **2.63** | **90.00** | **0.010** | **9.00** | **3.43** |
| ***SF*** |  | **2.04** | **90.00** | **0.044** | **7.00** | **3.43** |
| *SM* |  | 1.85 | 90.00 | 0.068 | 6.79 | 3.67 |
| **Order** | **6.2** |  | **2** | **0.045** |  |  |
| ***Day 2*** |  | **2.42** | **121.25** | **0.017** | **3.57** | **1.48** |
| *Day 3* |  | 0.96 | 121.25 | 0.730 | 1.41 | 1.48 |
| REMOVED TERMS | *Χ*^2^ |  | d.f. | p |  |  |
| Group size x Treatment | 8.22 |  | 2 | 0.061 |  |  |
| Group size | 0.09 |  | 1 | 0.766 |  |  |

**table S13** – Effect of subordinate female ability to observe the intrusion and/or participate in territorial defence (Experiment II) on post-intrusion changes in number of affiliative behaviours (a) displayed at group members and (b) received from group members.

| **a. Change in within-group affiliation displayed** | | | | | | |
| --- | --- | --- | --- | --- | --- | --- |
| Subject: Group: 24.26 ± 4.93; Group: 16.92 ± 4.11; Residual: 104.71 ± 10.23 | | | | | | |
| FINAL MODEL | *Χ*^2^ | t-value | d.f. | p | estimate | s.e. |
| Intercept |  | -0.25 | 60.21 | 0.806 | -0.67 | 2.70 |
| **Treatment** | **6.46** |  | **2** | **0.040** |  |  |
| *Uncooperative* |  | -1.47 | 96.00 | 0.145 | -3.04 | 2.07 |
| ***Unaware*** |  | **-2.55** | **96.00** | **0.013** | **-5.27** | **2.07** |
| **Individual category** | **11.64** |  | **3** | **0.009** |  |  |
| *DM* |  | -1.43 | 33.70 | 0.163 | -4.31 | 3.02 |
| ***SF*** |  | **2.03** | **33.70** | **0.050** | **6.13** | **3.02** |
| *SM* |  | 0.75 | 35.00 | 0.460 | 2.45 | 3.27 |
| REMOVED TERMS | *Χ*^2^ |  | d.f. | p |  |  |
| Group size x Treatment | 2.19 |  | 2 | 0.335 |  |  |
| Treatment x Individual category | 5.20 |  | 6 | 0.519 |  |  |
| Group size | 0.14 |  | 1 | 0.709 |  |  |
| Order | 3.23 |  | 2 | 0.199 |  |  |
| **b. Change in within-group affiliation received** | | | | | | |
| Subject: Group: 0.00 ± 0.00; Group: 26.65 ± 5.16; Residual: 53.03 ± 7.28 | | | | | | |
| FINAL MODEL | *Χ*^2^ | t-value | d.f. | p | estimate | s.e. |
| Intercept |  | -0.34 | 73.85 | 0.737 | -0.89 | 2.63 |
| Treatment |  |  |  |  |  |  |
| *Uncooperative* |  |  |  |  | -5.80 | 2.86 |
| *Unaware* |  |  |  |  | -6.91 | 2.86 |
| Individual category |  |  |  |  |  |  |
| *DM* |  |  |  |  | -2.15 | 2.86 |
| *SF* |  |  |  |  | 1.69 | 2.86 |
| *SM* |  |  |  |  | -2.31 | 3.08 |
| **Treatment x Individual category** | **14.16** |  | **6** | **0.025** |  |  |
| Uncooperative treatment x Individual category | |  |  |  |  |  |
| *DM* |  | 1.39 | 121.25 | 0.167 | 5.62 | 4.04 |
| *SF* |  | -1.38 | 121.25 | 0.734 | -1.38 | 4.04 |
| ***SM*** |  | **2.13** | **121.25** | **0.035** | **9.24** | **4.33** |
| Unaware treatment x Individual category | |  |  |  |  |  |
| *DM* |  | 1.79 | 121.25 | 0.076 | 7.23 | 4.04 |
| *SF* |  | -1.13 | 121.25 | 0.248 | -4.69 | 4.04 |
| *SM* |  | 1.20 | 121.25 | 0.233 | 5.20 | 4.34 |
| **Order** | **6.42** |  | **2** | **0.040** |  |  |
| ***Day 2*** |  | **2.42** | **121.25** | **0.017** | **3.57** | **1.48** |
| *Day 3* |  | 0.96 | 121.25 | 0.730 | 1.41 | 1.48 |
| REMOVED TERMS | *Χ*^2^ |  | d.f. | p |  |  |
| Group size x Treatment | 3.45 |  | 2 | 0.179 |  |  |
| Group size | 0.15 |  | 1 | 0.701 |  |  |

**table S14** – – Effect of subordinate female ability to observe the intrusion and/or participate in territorial defence (Experiment II) on changes in the number of (a) DF aggressive behaviours directed at the SF, (b) DF affiliative behaviours directs at SF, (c) SF submissive behaviours directed at the DF, and (d) SF affiliative behaviours directed at the DF.

| **a. Change in DF aggression directed at SF** | | | | | | |
| --- | --- | --- | --- | --- | --- | --- |
| Subject: 0.00 ± 0.00; Residual: 46.72 ± 6.84 | | | | | | |
| FINAL MODEL | *Χ*^2^ | t-value | d.f. | p | estimate | s.e. |
| Intercept |  | -1.29 | 38.00 | 0.205 | -1.41 | 1.10 |
| REMOVED TERMS | *Χ*^2^ |  | d.f. | p |  |  |
| Group size x Treatment | 1.09 |  | 2 | 0.581 |  |  |
| Group size | 0.09 |  | 1 | 0.765 |  |  |
| Order | 4.57 |  | 2 | 0.102 |  |  |
| Treatment | 5.08 |  | 2 | 0.079 |  |  |
| **b. Change in DF affiliation directed at SF** | | | | | | |
| Subject: 1.19 ± 1.09; Residual: 28.45 ± 5.33 | | | | | | |
| FINAL MODEL | *Χ*^2^ | t-value | d.f. | p | estimate | s.e. |
| Intercept |  | 1.73 | 35.88 | 0.092 | 2.62 | 1.51 |
| **Treatment** | **7.33** |  | **2** | **0.026** |  |  |
| *Uncooperative* |  | -1.62 | 24.00 | 0.119 | -3.39 | 2.09 |
| ***Unaware*** |  | **-2.76** | **24.00** | **0.011** | **-5.77** | **2.09** |
| REMOVED TERMS | *Χ*^2^ |  | d.f. | p |  |  |
| Group size x Treatment | 0.88 |  | 2 | 0.644 |  |  |
| Group size | 0.51 |  | 1 | 0.477 |  |  |
| Order | 3.9 |  | 2 | 0.142 |  |  |
| **c. Change in SF submission directed at DF** | | | | | | |
| Subject: 19.26 ± 4.39; Residual: 32.58 ± 5.71 | | | | | | |
| FINAL MODEL | *Χ*^2^ | t-value | d.f. | p | estimate | s.e. |
| Intercept |  | 1.89 | 28.21 | 0.069 | 3.77 | 2.00 |
| **Order** | **8.76** |  | **2** | **0.013** |  |  |
| *Day 2* |  | -1.34 | 24 | 0.193 | -3.00 | 2.24 |
| ***Day 3*** |  | **-3.09** | **24.00** | **0.005** | **-6.92** | **2.24** |
| REMOVED TERMS | *Χ*^2^ |  | d.f. | p |  |  |
| Group size x Treatment | 0.44 |  | 2 | 0.802 |  |  |
| Group size | 1.68 |  | 1 | 0.195 |  |  |
| Treatment | 0.17 |  | 2 | 0.917 |  |  |
| **d. Change in SF affiliation directed at DF** | | | | | | |
| Subject: 0.00 ± 0.00; Residual: 35.62 ± 5.97 | | | | | | |
| FINAL MODEL | *Χ*^2^ | t-value | d.f. | p | estimate | s.e. |
| Intercept |  | 1.63 | 36.00 | 0.113 | 2.69 | 1.66 |
| **Treatment** | **6.33** |  | **2** | **0.042** |  |  |
| *Uncooperative* |  | -0.26 | 36.00 | 0.794 | -0.62 | 2.34 |
| ***Unaware*** |  | **-2.30** | **36.00** | **0.027** | **-5.39** | **2.34** |
| REMOVED TERMS | *Χ*^2^ |  | d.f. | p |  |  |
| Group size x Treatment | 0.40 |  | 2 | 0.820 |  |  |
| Order | 0.95 |  | 2 | 0.620 |  |  |
| Group size | 1.03 |  | 1 | 0.310 |  |  |

a)


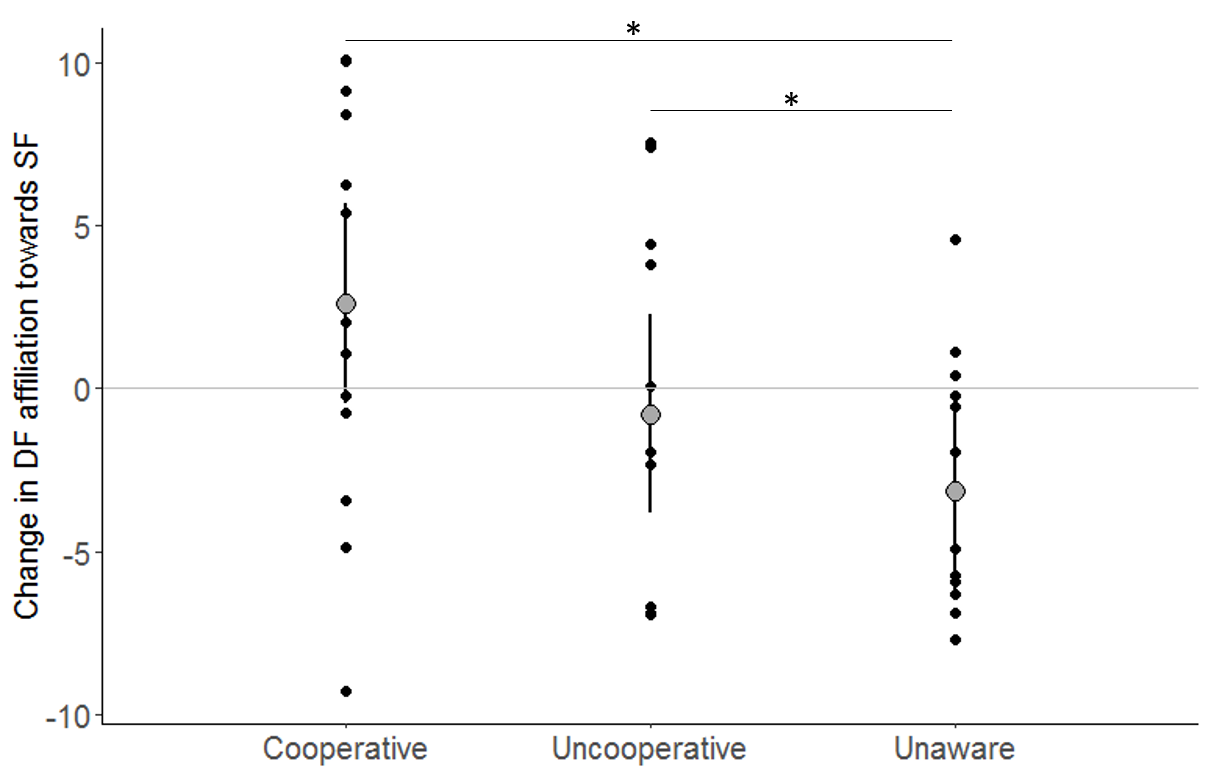


b)


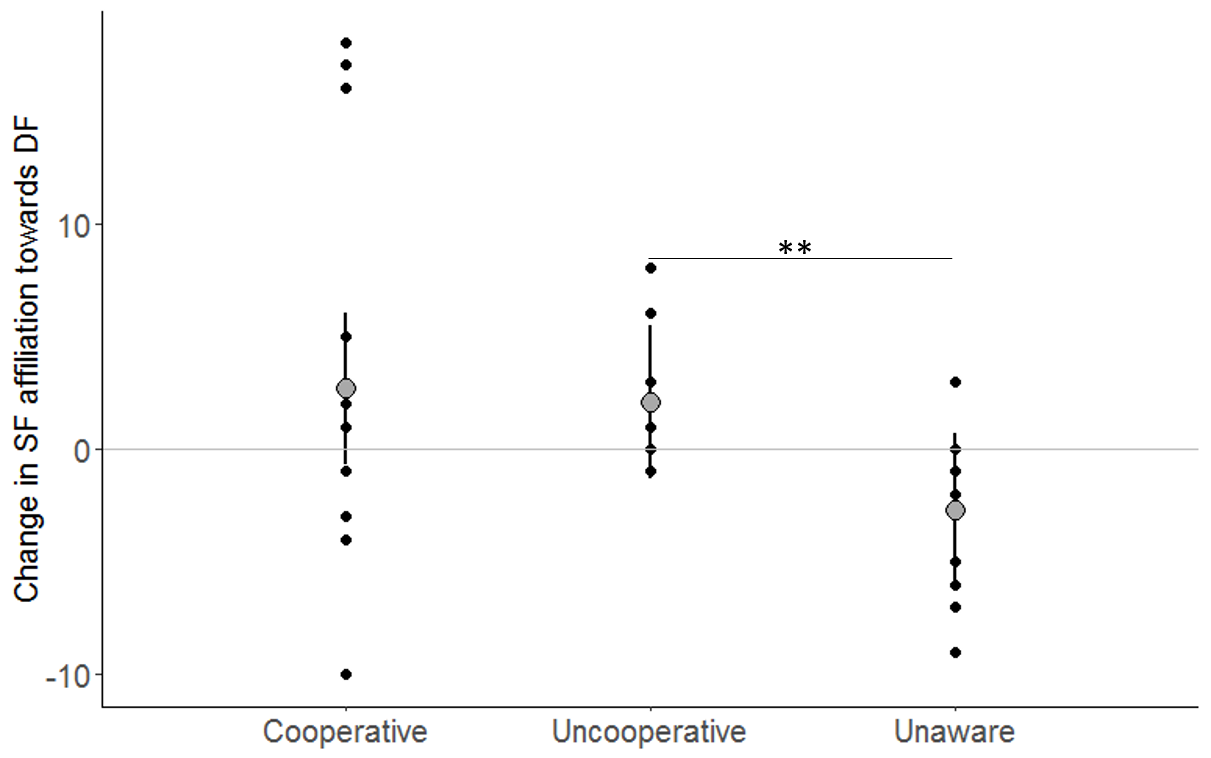


**Figure S1 -** Post-intrusion change in frequency of (a) dominant female (DF) affiliation directed at subordinate females (SFs) and (b) SF affiliation directed at DFs depending on variation in subordinate female presence or absence and contribution to territorial defence (Experiment II). Figures show fitted values (mean ± 95% confidence intervals) and partial residuals (black dots) from LMMs presented in table S14. Significant treatment differences highlighted. * p < 0.05, ** p < 0.01.
